# Efficacy of intraoperative transesophageal echocardiography in a case of protamine shock during transcatheter aortic valve implantation

**DOI:** 10.1186/s40981-016-0053-6

**Published:** 2016-10-10

**Authors:** Akihisa Kataoka, Yusuke Watanabe, Shutaro Seki, Shintaro Takamura, Hirofumi Hioki, Hiroyuki Kyono, Shigehito Sawamura, Ken Kozuma

**Affiliations:** 1 Department of Medicine, Division of Cardiology, Teikyo University, 2-11-1 Kaga, Itabashi-ku, Tokyo, 173-8606 Japan; 2 Department of Anesthesia, Teikyo University, 2-11-1 Kaga, Itabashi-ku, Tokyo, 173-8606 Japan

**Keywords:** Transcatheter aortic valve implant, Transesophageal echocardiography, Protamine shock, Acute pulmonary hypertension

## Abstract

Here, we report the case of a patient who developed protamine shock during a transcatheter aortic valve implant (TAVI) procedure, which was diagnosed by intraoperative transesophageal echocardiography (TEE). A 77-year-old man with symptomatic severe aortic stenosis and reduced left ventricular (LV) function underwent TAVI under general anesthesia. During the procedure, a transcatheter heart valve (THV) was deployed via the transfemoral approach, without any other major complications. The entire device system was then removed, and protamine sulfate was administered intravenously in 2 min.

Two minutes after the protamine administration, severe hypotension occurred. TEE did not reveal THV malfunction or any other major complications. However, comparison of the TEE image obtained before protamine administration and that obtained 2 min after protamine administration showed right ventricular (RV) dilatation, RV free wall motion abnormality, and LV volume reduction, without any electrocardiographic changes. We diagnosed this as protamine shock and bolus infusions of phenylephrine and norepinephrine were administered, and chest compressions were initiated immediately. After 1 min, hypotension as well as the right and left ventricular size and dysfunction immediately reverted to baseline. The severe systemic hypotension resolved as well. Thereafter, he recovered from anesthesia without other complications.

This case showed the clinical features of protamine shock with acute pulmonary hypertension. The TEE images, in this case, should be a reminder for all doctors who perform intraoperative TEE for patient monitoring when they perform procedures to treat structural heart diseases.

**Electronic supplementary material:**

The online version of this article (doi:10.1186/s40981-016-0053-6) contains supplementary material, which is available to authorized users.

## Background

Protamine is the mainstay drug for heparin neutralization during cardiac surgery, structural heart disease (SHD) intervention, or coronary intervention [1]. Transcatheter aortic valve implantation (TAVI), a common minimally invasive approach for treating severe aortic valve stenosis (AS), is currently performed worldwide in elderly and high-risk patients [2]. Here, we present a case of protamine shock during a TAVI procedure in which many other causes of hypotension were considered and intraoperative transesophageal echocardiography (TEE) was useful for arriving at the diagnosis.

## Case presentation

**Fig. 1:**
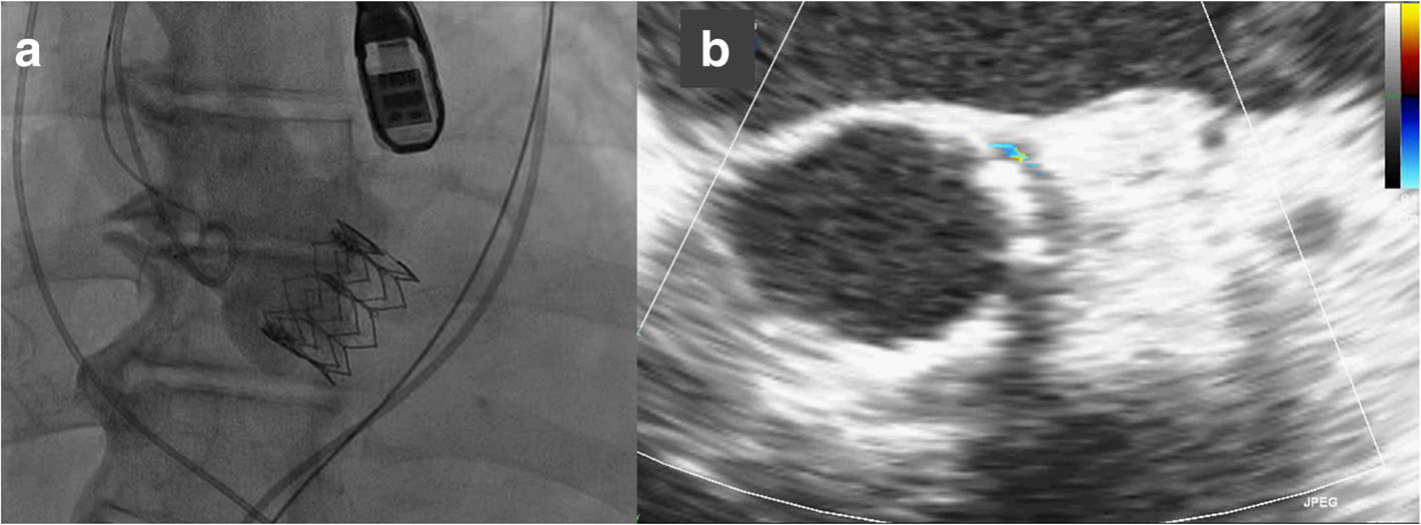
Aortography and TEE images after THV implantation. Aortography (**a**) and short-axis intraoperative TEE (**b**) images of the transcatheter heart valve (SAPIEN XT 26 mm) showing only trivial paravalvular leakage, without any other complications. *THV* transcatheter heart valve

A 77-year-old man with symptomatic severe AS and reduced left ventricular (LV) function underwent TAVI under general anesthesia. During the procedure, dobutamine (2–5 μg/kg/min) and nicorandil (3 mg/h) were continuously infused to maintain hemodynamic stability. A transcatheter heart valve (THV) (SAPIEN XT 26 mm) was deployed via the transfemoral approach with an electrocardiographic change in the left bundle branch block (LBBB), without any other major complications (Fig. 1a, b). Thereafter, the entire device system was removed, and protamine sulfate (60 mg) was administered intravenously in 2 min to achieve appropriate hemostasis.

**Fig. 2:**
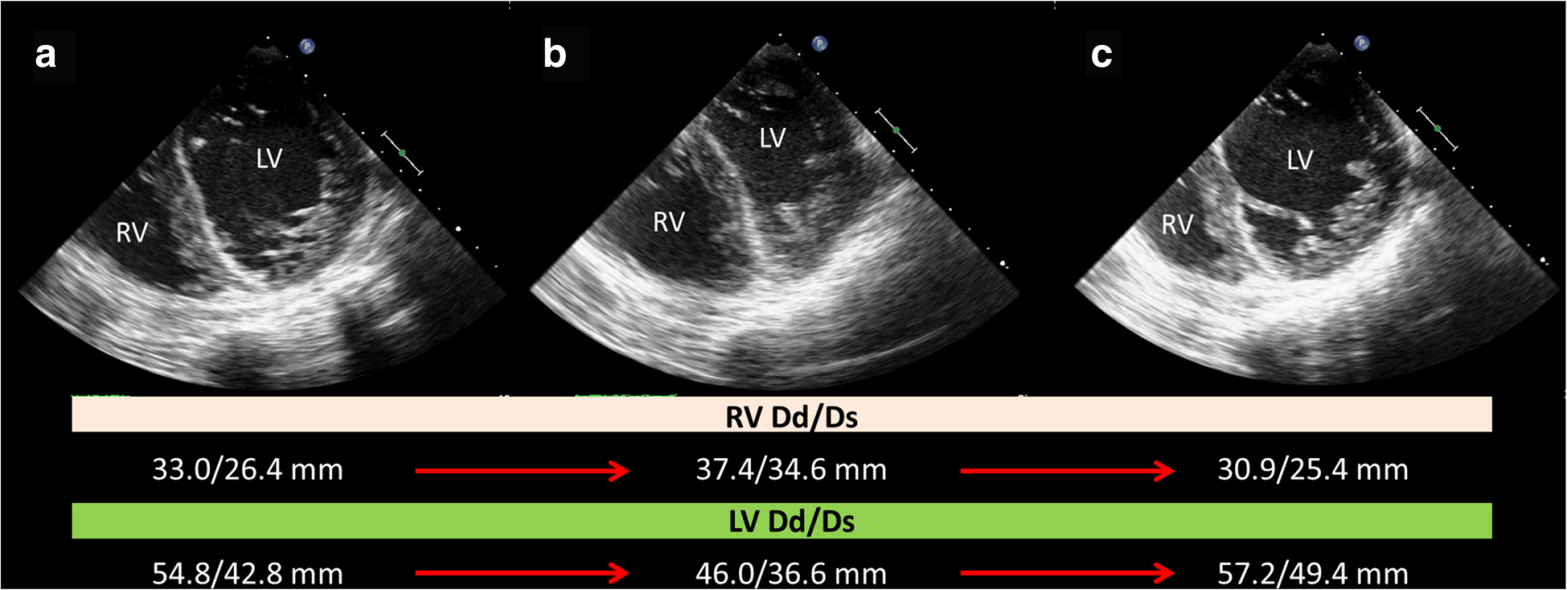
Mid-esophageal four-chamber TEE images collected during the procedure. **a** TEE image after THV deployment showing normal RV size (RV Dd/Ds = 33.0/26.4 mm). **b** TEE image collected 2 min after protamine administration demonstrating RV dilatation (*yellow arrow*) (RV Dd/Ds = 37.4/34.6 mm) and left ventricular (LV Dd/Ds = 46.0/36.6 mm) volume reduction. Severe systemic hypotension was observed at the same time. **c** TEE image after 1 min of chest compressions (approximately 5 min after protamine administration) demonstrating that the RV and LV size and wall motion had returned to baseline (RV Dd/Ds = 30.9/25.4 mm, LV Dd/Ds = 57.2/49.4 mm). *Dd* dimension end-diastole, *Ds* dimension end-systole, *LV* left ventricle, *RV* right ventricle, *TEE* transesophageal echocardiography


Two minutes after the protamine administration, severe hypotension occurred (30/10 mmHg). TEE did not reveal THV malfunction or any other major complications; however, comparison of the TEE image obtained before the protamine administration (Fig. 2a, Additional file 1: Video 1) and that obtained 2 min after the protamine administration showed right ventricular (RV) dilatation, RV free wall motion abnormality, and LV volume reduction (Fig. 2b, Additional file 2: Video 2) without any electrocardiographic changes, indicative of a persistent LBBB, and pulse oximetry changes. We diagnosed this as protamine shock, and then, three bolus infusions of phenylephrine (0.1 mg) followed by a bolus infusion of norepinephrine (50 μg) were immediately administered, and chest compressions were initiated simultaneously. After 1 min, hypotension as well as the right and left ventricular size and dysfunction immediately reverted to baseline (Fig. 2c, Additional file 3: Video 3). The severe systemic hypotension resolved as well. Thereafter, he recovered from anesthesia without other complications and blood gas abnormalities.

Additional file 2: Video 2. TEE moving image 2 min after protamine administration. RV dilatation, RV free wall motion abnormality, and LV volume reduction in the mid-esophageal four-chamber TEE view 2 min after protamine administration. LV, left ventricle. (AVI 5923 kb)


### Discussion

This case showed the clinical features of protamine shock by acute pulmonary hypertension (APH), a rare cause of hypotension during the TAVI procedure. The transfemoral approach TAVI procedure has several well-known causes of systemic hypotension such as access site bleeding, cardiac tamponade, aortic root rupture, coronary obstruction, aortic regurgitation, mitral regurgitation, and THV malfunction [3]. TEE images can be used to assess these major complications immediately [4]. In this case, we found rare images showing RV dilatation and free wall motion abnormality followed by an LV volume reduction that suggested the existence of APH. These images indicated several differential diagnoses such as protamine-induced APH, acute pulmonary embolism, and RV infarction. First, we excluded the possibility of RV infarction due to coronary obstruction. Although an LBBB appeared after THV deployment, the normal RV free wall motion persisted prior to the development of hypotension. In addition, there were no electrocardiographic changes around the time of the severe hypotension. We ultimately diagnosed protamine-induced APH rather than a pulmonary embolism, considering of the time course in which the RV dysfunction recovered after only 1 min of chest compressions.

Protamine remains the mainstay drug for heparin neutralization during coronary intervention or cardiac surgery, and it occasionally causes transient hypotension known as “protamine shock” in clinical settings [5]. Protamine shock has three different causes: APH, anaphylactoid reaction, and histamine release followed by rapid injection [5, 6]. APH is a clinical diagnosis and uncommon complication of protamine administration which rarely occurs with an incidence of 0.06 % [5, 7]. However, it is severe and life threatening. Multiple immunological and non-immunological processes can trigger acute pulmonary vasoconstriction, which increases pulmonary artery pressure, thereby resulting in RV dilatation and dysfunction followed by an LV volume reduction due to poor LV filling and, consequently, systemic hypotension [6–8]. The TEE images, in this case, were able to capture this pathognomonic biventricular failure, which helped us with the diagnosis.

There are several suggested potential risk factors for protamine shock, including a history of vasectomy, previous protamine exposure, vertebrate fish allergy, and preexisting pulmonary hypertension [5, 9]. It is interesting to note that, despite the fact that our patient did not have any risk factors and that we carefully and slowly injected the protamine, he experienced protamine shock. Fortunately, his blood pressure recovered within 5 min, without the need for an artificial heart-lung machine or percutaneous cardiopulmonary support system. This result might be due to the fact that the amount of protamine sulfate was only one third (60 mg) of that used in general cardiac surgery. The numbers of TAVI and SHD intervention procedures, which require the use of protamine, performed in Japan have drastically increased in recent years. Accordingly, clinicians should carefully perform intraoperative TEE monitoring for protamine shock.

## Conclusions

Here, we reported the case of a patient who developed protamine shock with APH during a TAVI procedure in which TEE was useful for making the diagnosis. The images obtained, in this case, should remind all doctors to perform intraoperative TEE monitoring during TAVI and SHD procedures whenever protamine is used.

## Additional files

Additional file 1: Video 1. TEE moving image after THV deployment. Normal RV size and RV free wall motion in the mid-esophageal four-chamber TEE view after THV deployment. RV, right ventricle; TEE, transesophageal echocardiography; THV, transcatheter heart valve. (AVI 3462 kb)

Additional file 3: Video 3. TEE moving image after 1 min of chest compressions. Recovered size and wall motion of RV and LV to baseline in the mid-esophageal four-chamber TEE view after 1 min of chest compressions. (AVI 2758 kb)

## Abbreviations

APH: Acute pulmonary hypertension
AS: Aortic valve stenosis
LBBB: Left bundle branch block
LV: Left ventricular
RV: Right ventricular
SHD: Structural heart disease
TAVI: Transcatheter aortic valve implantation
TEE: Transesophageal echocardiography
THV: Transcatheter heart valve
